# A Case of Refractory Thrombocytopenia in Pregnancy

**DOI:** 10.7759/cureus.113010

**Published:** 2026-07-20

**Authors:** David B Joseph, Andrea Bequest, Pinky Jha, Hetvi Patel

**Affiliations:** 1 Department of Family Medicine, Medical College of Wisconsin, Milwaukee, USA; 2 Department of Internal Medicine, Medical College of Wisconsin, Milwaukee, USA

**Keywords:** corticosteroids, immune thrombocytopenia, intravenous immunoglobulin, maternal-fetal medicine, pregnancy, thrombocytopenia

## Abstract

Immune thrombocytopenia is an acquired autoimmune disorder characterized by isolated thrombocytopenia due to antibody-mediated platelet destruction and impaired platelet production. Thrombocytopenia in pregnancy is common, but severe thrombocytopenia requires prompt evaluation to distinguish benign gestational thrombocytopenia from immune thrombocytopenia, hypertensive disorders of pregnancy, hemolysis, elevated liver enzymes, low platelet count syndrome, and thrombotic microangiopathies. We present the case of a 19-year-old gravida 1 para 0 patient with sickle cell trait and iron deficiency anemia who was initially diagnosed with pregnancy-associated immune thrombocytopenia in the first trimester after presenting with severe thrombocytopenia, with a platelet count of 9 × 10³/µL. She initially responded to intravenous immunoglobulin and corticosteroids, with platelet recovery to 107 × 10³/µL during hospitalization and 264 × 10³/µL shortly after discharge. However, despite outpatient hematology follow-up and prednisone therapy, her platelet count progressively declined. She was readmitted at 22 weeks and 5 days of gestation after routine obstetric laboratory testing showed relapsing severe thrombocytopenia with platelets of 15 × 10³/µL. The patient was otherwise asymptomatic, aside from intermittent blood-tinged nasal discharge and mild vaginal spotting a few days before presentation that resolved spontaneously. She was normotensive and had no evidence of hemolysis, renal dysfunction, significant hepatic dysfunction, or coagulopathy. Hematology and maternal-fetal medicine were consulted, and she was treated with intravenous immunoglobulin with post-treatment platelet improvement to 53 × 10³/µL. She was discharged on a prednisone taper with plans for scheduled outpatient intravenous immunoglobulin and close follow-up in a combined hematology and maternal-fetal medicine clinic. This case highlights the importance of a structured, multidisciplinary approach to severe thrombocytopenia in pregnancy and the need to exclude pregnancy-specific and life-threatening alternative etiologies before confirming immune thrombocytopenia.

## Introduction

Thrombocytopenia, commonly defined as a platelet count below 150 × 10⁹/L, is the second most common hematologic abnormality in pregnancy after anemia and is reported in approximately 5%-10% of pregnancies [1-5]. Although many cases are detected incidentally during routine prenatal laboratory testing, thrombocytopenia in pregnancy has a broad differential diagnosis that includes benign gestational thrombocytopenia; immune thrombocytopenia (ITP); hypertensive disorders of pregnancy, hemolysis, elevated liver enzymes, low platelet count (HELLP) syndrome; thrombotic thrombocytopenic purpura; hemolytic uremic syndrome; disseminated intravascular coagulation; medication-related thrombocytopenia; infection-associated thrombocytopenia; and inherited platelet disorders [1-4,6,7]. Establishing the correct diagnosis is clinically important because these etiologies differ substantially in maternal risk, fetal risk, treatment strategy, timing of delivery, and anesthesia planning.

The timing of onset and severity of thrombocytopenia are key diagnostic clues emphasized in the obstetric and hematology literature [1,2,6,7]. Gestational thrombocytopenia is the most common cause of thrombocytopenia in pregnancy and typically develops in the late second or third trimester. It is usually mild, asymptomatic, and not associated with maternal bleeding or neonatal thrombocytopenia; platelet counts generally remain above 100 × 10⁹/L and normalize after delivery [1,3,6,7]. Therefore, thrombocytopenia that begins in the first trimester, platelet counts below 100 × 10⁹/L, rapidly declining platelet counts, or mucocutaneous bleeding should prompt evaluation for alternative causes, including ITP and thrombotic microangiopathies [1-3,6].

Pregnancy-specific disorders must be considered urgently when thrombocytopenia is accompanied by hypertension, proteinuria, hemolysis, renal dysfunction, or hepatic injury. Preeclampsia and HELLP syndrome are among the most important obstetric causes because they can progress quickly and may require delivery as definitive management [1,2,6,7]. Thrombotic microangiopathies such as thrombotic thrombocytopenic purpura and complement-mediated hemolytic uremic syndrome may overlap clinically with HELLP syndrome but require distinct treatment. As a result, evaluation of severe thrombocytopenia in pregnancy should include blood pressure assessment, peripheral smear review when available, hemolysis testing, renal function, liver enzymes, bilirubin, coagulation studies, and careful review of medication and infection exposures [1-3,6].

ITP is an acquired autoimmune disorder characterized by antibody-mediated platelet destruction and impaired platelet production. It may predate pregnancy or first become apparent during gestation, and it is an important cause of early-onset or severe isolated thrombocytopenia [2,4,5]. Pregnancy adds management complexity because clinicians must balance maternal bleeding risk against fetal safety, medication adverse effects, procedural platelet thresholds, delivery planning, and the possibility of neonatal thrombocytopenia from transplacental passage of antiplatelet antibodies [1,2,4-7].

Current management of ITP in pregnancy is individualized and multidisciplinary. Treatment is generally reserved for patients with clinically significant bleeding, very low platelet counts, or the need to achieve procedural or delivery-related platelet thresholds [1,2,4,5,7]. First-line therapies include corticosteroids and intravenous immunoglobulin (IVIG), while refractory or relapsing disease may require repeated IVIG, treatment escalation, or individualized second-line therapy in consultation with hematology and maternal-fetal medicine [2,4,5]. We present a case of relapsing severe pregnancy-associated ITP requiring repeated IVIG and corticosteroid therapy, highlighting the importance of a structured diagnostic approach, exclusion of pregnancy-specific mimics, and coordinated multidisciplinary care.

## Case presentation

A 19-year-old gravida 1 para 0 patient with sickle cell trait and no prior history of thrombocytopenia presented for her first prenatal visit at eight weeks and three days of gestation. She was clinically well and denied bruising, petechiae, epistaxis, vaginal bleeding, or other bleeding symptoms. Routine prenatal laboratory testing showed a platelet count of 36 × 10³/µL, hemoglobin of 9.5 g/dL, and mean corpuscular volume of 68 fL. Repeat testing confirmed a platelet count of approximately 40 × 10³/µL. For comparison, platelet counts before pregnancy had been normal at 146-157 × 10³/µL. Iron studies demonstrated iron deficiency, with a ferritin of 10 ng/mL and transferrin saturation of 7%.

She underwent an expedited hematology evaluation at approximately nine weeks of gestation. At that visit, her platelet count was 41 × 10³/µL, hemoglobin was 9.2 g/dL, and mean corpuscular volume was 69 fL. She remained normotensive and had no active bleeding. Renal and hepatic function, prothrombin time, partial thromboplastin time, bilirubin, folate, and vitamin B12 levels were within reference ranges. Haptoglobin was not decreased, and the available laboratory evaluation did not support hemolysis, disseminated intravascular coagulation, preeclampsia, HELLP syndrome, or thrombotic microangiopathy. The pertinent diagnostic evaluation is summarized in Table 1. Given the early gestational onset, platelet count well below 100 × 10³/µL, previously normal platelet count, and absence of an alternative cause, hematology concluded that the presentation was most consistent with pregnancy-associated ITP. Weekly platelet monitoring was initiated.

**Table 1:** Longitudinal platelet, hemoglobin, and treatment course through 24 weeks’ gestation. An em dash indicates that the value was not documented in the supplied record for that date. ITP: immune thrombocytopenia; CBC: complete blood count; IV: intravenous; IVIG: intravenous immunoglobulin; MCV: mean corpuscular volume; w: weeks; d: days

Gestational age	Platelet count (10³/µL)	Hemoglobin (g/dL)	MCV (fL)	Clinical context	Treatment/Follow-up
Before pregnancy (2024)	157	10.8	82	Baseline platelet count was normal	No ITP treatment
8 w 3 d	36	9.5	68	First prenatal CBC; no bleeding, bruising, or petechiae	Aspirin held; urgent hematology referral; oral iron started
8 w 4 d	40	9.4	69	Ferritin 10 ng/mL; transferrin saturation 7%; smear without significant schistocytosis	Bleeding precautions; repeat testing
9 w 4 d	41	9.2	69	Benign hematology consultation; no active bleeding	Weekly CBC; observation because platelets remained >30
10 w 5 d	9 (11 on repeat)	9.5	69	Hospitalized for nausea/vomiting; no active bleeding	IVIG 1 g/kg plus prednisone ~1 mg/kg/day
10 w 6 d	55, then 73	8.4, then 8.9	70	Early response after IVIG and corticosteroids	Continued prednisone
11 w 0 d	107	8.2	71	Clinically stable for discharge	Prednisone taper; outpatient hematology follow-up
11 w 3 d	264	9.0	70	Peak outpatient response	Continued steroid taper
12 w 4 d	85	8.7	72	Asymptomatic decline during taper	Prednisone continued at 40 mg; weekly CBC
14 w 2 d	52	—	—	Further decline	Weekly CBC; prednisone adjusted
15 w 3 d	113	—	—	No bleeding	Taper restarted: 30 mg, then 20 mg, then 10 mg weekly
16 w 2 d	93	8.4	—	Clinically stable	Continued taper and monitoring
17 w 4 d	49	8.0	73	Platelet decline and persistent iron deficiency anemia	Prednisone maintained at 20 mg; intravenous iron arranged
18 w 0 d	—	—	—	Iron infusion; mild transient pruritus	Ferumoxytol 1,020 mg IV; infusion rate slowed
22 w 5 d	15 outpatient; 16 admission	10.9	78	Relapse within the same first pregnancy; no active bleeding	IVIG 1 g/kg; prednisone 20 mg initially continued
22 w 6 d	25	10.1	—	Early post-IVIG response	Continued inpatient monitoring; outpatient IVIG authorization initiated
23 w 0 d	53	9.5	81	Improved and discharged	Prednisone 10 mg for 1 week, then stop; IVIG approximately every 2 weeks; weekly CBC
23 w 5 d	156	—	—	Marked outpatient response after the second IVIG-treated episode	Continued weekly monitoring and planned scheduled IVIG
24 w 3 d	Most recent: 156	—	—	Clinically well; no bleeding; prednisone completed 3 days earlier	Ongoing benign hematology and maternal-fetal medicine follow-up

At 10 weeks and 5 days of gestation, she was admitted with nausea and vomiting and was found to have progression to profound thrombocytopenia, with platelet counts of 9-11 × 10³/µL. Hemoglobin was 9.5 g/dL with persistent microcytosis. She remained normotensive, and repeat renal, hepatic, coagulation, and hemolysis studies remained reassuring, as shown in Table 1. Because the platelet count had fallen below the treatment threshold, she received IVIG at 1 g/kg and prednisone at approximately 1 mg/kg daily, followed by a planned taper. Her platelet count increased to 55-73 × 10³/µL the following day, reached 107 × 10³/µL by discharge, and increased to 264 × 10³/µL on outpatient testing three days later. The relationship between platelet count, hemoglobin, corticosteroid therapy, and IVIG is presented chronologically in Table 2.

**Table 2:** Diagnostic evaluation at the initial severe presentation and relapse. Values reflect the clinically relevant diagnostic workup documented during the first-trimester evaluation and the 22-week relapse. HELLP: hemolysis, elevated liver enzymes, low platelet count; HIV: human immunodeficiency virus

Test (unit)	Initial evaluation/First severe presentation (8–10 weeks)	Relapse (22 weeks 5 days)	Reference range	Interpretation
Platelet count (10³/µL)	36–41 initially; nadir 9 (11 on repeat)	15 outpatient; 16 on admission	150–350	Profound isolated thrombocytopenia
Hemoglobin (g/dL)	9.2–9.5	10.9	12.0–15.0	Anemia was microcytic and iron deficient
Mean corpuscular volume (fL)	68–69	78	80–100	Microcytosis improved after iron treatment
Creatinine (mg/dL)	0.68–0.75	0.57	0.50–1.10	Preserved renal function
Aspartate aminotransferase (U/L)	18–20	35	<35	No clinically significant hepatic injury
Alanine aminotransferase (U/L)	16–18	32	<30	Minimal elevation without HELLP pattern
Alkaline phosphatase (U/L)	68–72	50	35–104	Within reference range
Total bilirubin (mg/dL)	0.2–0.3	<0.2	0.2–1.2	No biochemical evidence of hemolysis
Direct bilirubin (mg/dL)	<0.1	<0.1	≤0.3	Normal
Lactate dehydrogenase (U/L)	Not elevated in the available workup	205	135–214	No evidence of microangiopathic hemolysis
Haptoglobin (mg/dL)	277–314	Not repeated	30–200	Elevated rather than decreased; hemolysis unlikely
Prothrombin time (seconds)	9.8–10.4	9.9	9.5–11.8	Normal
International normalized ratio	0.9	0.9	0.9–1.2	Normal
Partial thromboplastin time (seconds)	25.8–26.2	25.6	23.0–30.0	Normal
Fibrinogen (mg/dL)	480–634	Not repeated	193–507	Not low; disseminated intravascular coagulation unlikely
Peripheral smear	0–2 schistocytes per oil-power field; no significant schistocytosis	No features suggesting microangiopathic hemolysis	None seen	Thrombotic microangiopathy not supported
Ferritin (ng/mL)/Transferrin saturation (%)	10/7 at diagnosis; 25.2/10 at hospitalization	Improved after intravenous iron	20–90/15–50	Confirmed concurrent iron deficiency anemia
Hepatitis B surface antigen, hepatitis C antibody, and HIV antigen/antibody	Nonreactive	No new exposure identified	Nonreactive	Secondary infectious causes not identified
Obstetric ultrasound	Viable intrauterine pregnancy at 8 weeks 3 days	Single living fetus, cephalic, anterior placenta without previa, normal amniotic fluid	Not applicable	Reassuring fetal assessment

During outpatient follow-up, the platelet count declined as prednisone was tapered, although it remained substantially improved from the initial nadir. Platelet counts were 85 × 10³/µL at approximately 12 weeks, 52 × 10³/µL at approximately 14 weeks, 113 × 10³/µL at 15 weeks, and 93 × 10³/µL at 16 weeks. The prednisone taper was therefore adjusted and intermittently held according to the platelet response. She continued to deny significant bleeding, bruising, or petechiae. Her concurrent iron deficiency anemia worsened, with hemoglobin declining to 8.0 g/dL by approximately 17 weeks, and she subsequently received intravenous iron. The hematologic course and associated treatments are summarized in Table 2.

Despite continued prednisone at 20 mg daily, her platelet count again progressively declined. At 22 weeks and 5 days of gestation, routine outpatient testing showed a platelet count of 15 × 10³/µL, prompting hospital admission for a relapse of profound thrombocytopenia within the same first pregnancy. She reported a prior minor episode of epistaxis and a small amount of vaginal spotting several days earlier, both of which had resolved. At admission, she denied active mucosal bleeding, petechiae, bruising, vaginal bleeding, abdominal pain, headache, or visual symptoms.

Her blood pressure was 110/76 mmHg, and she remained normotensive throughout the hospitalization. Laboratory testing showed a platelet count of 16 × 10³/µL, hemoglobin of 10.9 g/dL, creatinine of 0.57 mg/dL, total bilirubin below 0.2 mg/dL, lactate dehydrogenase of 205 U/L, international normalized ratio of 0.9, and partial thromboplastin time of 25.6 seconds. There was no evidence of hemolysis, renal dysfunction, clinically significant hepatic injury, or coagulopathy. These findings are detailed in Table 1. The early onset, isolated platelet decline, prior treatment response, and absence of hypertension or microangiopathic features supported relapse of ITP rather than gestational thrombocytopenia, preeclampsia, HELLP syndrome, or thrombotic microangiopathy.

Obstetric ultrasound showed a single viable fetus consistent with 22 weeks and 5 days of gestation, with normal amniotic fluid, an anterior placenta without previa, and no acute obstetric abnormality. Maternal-fetal medicine and hematology were consulted. She received another dose of IVIG at 1 g/kg. Her platelet count increased to 25 × 10³/µL the following morning and to 53 × 10³/µL on subsequent testing, as shown in Table 2.

She was discharged on hospital day two with instructions to taper and discontinue prednisone, monitor closely for bleeding, and continue frequent complete blood count surveillance. Because of the initial response followed by relapse during corticosteroid tapering, hematology recommended scheduled outpatient IVIG approximately every two weeks in coordination with maternal-fetal medicine. By approximately 24 weeks of gestation, her platelet count had improved to 156 × 10³/µL following IVIG, and she remained clinically stable without active bleeding. Ongoing management included weekly platelet monitoring and combined hematology and maternal-fetal medicine follow-up, as summarized in Table 2.

## Discussion

ITP is an acquired autoimmune disorder characterized by isolated thrombocytopenia due to accelerated immune-mediated platelet destruction and impaired platelet production. During pregnancy, thrombocytopenia affects approximately 5%-10% of patients, but most cases are due to benign gestational thrombocytopenia [1,3,6,7]. The timing of onset, severity of thrombocytopenia, presence or absence of bleeding, blood pressure pattern, and associated laboratory abnormalities are central to differentiating ITP from other pregnancy-associated causes of thrombocytopenia [1-3,6,7].

Gestational thrombocytopenia is the most common cause of thrombocytopenia in pregnancy and typically occurs in the late second or third trimester. It is generally mild, asymptomatic, and not associated with maternal or fetal complications. Platelet counts below 100 × 10⁹/L, thrombocytopenia in the first trimester, or clinically significant bleeding should prompt evaluation for alternative causes [1-3,6,7]. In contrast, ITP may occur at any gestational age, may precede pregnancy, and can produce severe thrombocytopenia requiring treatment.

The pathophysiology of ITP involves autoantibodies directed against platelet surface glycoproteins, leading to splenic platelet destruction and impaired megakaryocyte platelet production. In pregnancy, physiologic hemodilution, increased platelet activation, and immune changes may further lower platelet counts in patients with preexisting or newly recognized ITP [2-4]. Patients may be asymptomatic or may present with mucocutaneous bleeding, petechiae, bruising, epistaxis, or, outside pregnancy, menorrhagia. Severe bleeding is uncommon but may occur with profound thrombocytopenia.

ITP in pregnancy remains a diagnosis of exclusion. A structured workup includes review of the timing and severity of thrombocytopenia, medication exposure, bleeding symptoms, blood pressure trends, peripheral smear when available, renal function, liver enzymes, bilirubin, lactate dehydrogenase, coagulation studies, and evaluation for infection or autoimmune disease when clinically indicated [1-3,6,7]. In this case, the platelet trend in Table 2 supported a relapsing pregnancy-associated ITP because thrombocytopenia was severe, began early in pregnancy, was isolated, and had previously responded to IVIG and corticosteroids. The absence of hypertension argued against preeclampsia. Preserved renal function, normal bilirubin, normal coagulation studies, and normal lactate dehydrogenase argued against HELLP syndrome, disseminated intravascular coagulation, and thrombotic microangiopathy as summarized in Table 1.

The patient’s sickle cell trait was not considered the cause of the platelet decline. She had previously normal platelet counts and no evidence of hemolysis, sickling complications, or splenic sequestration. The associated anemia was instead supported as iron deficiency by marked microcytosis, low ferritin, and low transferrin saturation, with improvement after intravenous iron (Tables 1, 2).

International nomenclature classifies ITP by duration as newly diagnosed during the first three months, persistent from three to 12 months, and chronic after 12 months [5]. By the 22-week relapse, approximately 14 weeks had elapsed from the first detection, fitting persistent ITP by duration.

Management of ITP in pregnancy aims to prevent clinically important bleeding and maintain platelet counts adequate for necessary procedures while minimizing maternal and fetal treatment effects. The mode of delivery should be based on obstetric indications rather than ITP alone. Platelet count informs whether additional therapy is needed to reach hemostatic thresholds for vaginal delivery, cesarean delivery, or neuraxial anesthesia [1-4,7]. First-line therapy includes corticosteroids and IVIG. Corticosteroids are commonly used because of efficacy and availability, but prolonged use during pregnancy may be associated with adverse effects such as hyperglycemia, hypertension, mood changes, and gastrointestinal symptoms. IVIG can produce a more rapid platelet response and is often used when urgent platelet increase is needed, when corticosteroids are ineffective, or when steroid-related toxicity is a concern [2-4].

Refractory or relapsing ITP in pregnancy is challenging because data on second-line therapies are limited. Options such as splenectomy, rituximab, azathioprine, and thrombopoietin receptor agonists may be considered in selected cases after individualized risk-benefit discussion with hematology and maternal-fetal medicine [2,4,5]. In this case, hematology recommended scheduled outpatient IVIG because the patient had a relapse with platelet decline despite prednisone and had demonstrated platelet response to IVIG, with the treatment-associated platelet changes summarized in Table 2.

Delivery planning requires coordination among obstetrics, hematology, anesthesia, and pediatrics. Platelet goals vary by clinical context, but commonly used thresholds include at least 30-50 × 10⁹/L for vaginal or cesarean delivery and higher thresholds, often approximately 70-80 × 10⁹/L, for neuraxial anesthesia [1-4,7]. The mode of delivery should generally be determined by obstetric indications rather than maternal platelet count alone. Because maternal antiplatelet antibodies can cross the placenta, neonates born to patients with ITP require platelet monitoring after delivery. Neonatal thrombocytopenia occurs in a minority of infants, and severe bleeding is rare, but early recognition is important [1,2,7].

This case illustrates several points. First, severe thrombocytopenia in pregnancy should not be assumed to be gestational thrombocytopenia, especially when platelet counts are below 100 × 10⁹/L or when the onset occurs early in pregnancy. Second, the longitudinal platelet pattern that was responsive to IVIG in Table 2, combined with normotension and the absence of hemolysis, renal dysfunction, coagulopathy, or significant liver injury pattern in Table 1, supported ITP over preeclampsia, HELLP syndrome, and thrombotic microangiopathy. Third, refractory or relapsing ITP requires multidisciplinary planning with hematology and maternal-fetal medicine to guide treatment, monitoring, and delivery planning.

## Conclusions

This case describes pregnancy-associated ITP first recognized during a 19-year-old patient’s first pregnancy. Thrombocytopenia was detected at 8 weeks and 3 days, progressed to a platelet nadir of 9 × 10³/µL at 10 weeks and 5 days, and improved after IVIG and prednisone. During outpatient corticosteroid tapering, the platelet count declined again, culminating in a relapse to 15-16 × 10³/µL at 22 weeks and 5 days within the same pregnancy. Repeat evaluation showed normotension and no evidence of hemolysis, renal dysfunction, significant hepatic dysfunction, or coagulopathy. A second course of IVIG increased the platelet count to 53 × 10³/µL before discharge and 156 × 10³/µL on follow-up. By 24 weeks, prednisone had been discontinued, and the patient remained under combined benign hematology and maternal-fetal medicine care with weekly laboratory monitoring and planned outpatient IVIG. This case highlights a severe relapsing pregnancy-associated ITP requiring IVIG and corticosteroid therapy. In short, this case emphasizes the importance of a structured diagnostic approach to thrombocytopenia in pregnancy, including careful exclusion of gestational thrombocytopenia, hypertensive disorders of pregnancy, HELLP syndrome, and thrombotic microangiopathies. Early hematology and maternal-fetal medicine involvement is essential for treatment selection, platelet monitoring, delivery planning, and neonatal risk assessment. Severe thrombocytopenia in pregnancy should prompt timely multidisciplinary evaluation to optimize maternal and fetal outcomes.
